# Avoiding stoma creation due to super-morbid obesity: A report of two surgical cases of colorectal cancer

**DOI:** 10.1016/j.ijscr.2023.109171

**Published:** 2023-12-17

**Authors:** Naoto Fujimoto, Takayuki Ogino, Norikatsu Miyoshi, Mamoru Uemura, Yuichiro Doki, Hidetoshi Eguchi

**Affiliations:** a Department of Gastroenterological Surgery, Graduate school of medicine, Osaka University, Suita, Osaka, Japan; b Therapeutics for Inflammatory Bowel Diseases, Graduate school of medicine, Osaka University, Suita, Osaka, Japan

**Keywords:** Obesity, Colorectal cancer, Stoma, Chemotherapy

## Abstract

**Introduction:**

A stoma is commonly created in patients with a high risk of anastomotic leakage. However, patients with obesity have a higher incidence of stoma-related complications, and the decision to create a stoma should be carefully considered. We report two cases of patients with colorectal cancer and super-morbid obesity wherein stoma creation was avoided.

**Presentation of cases:**

Case 1 involved a 52-year-old male patient with a body mass index (BMI) of 41.8 kg/m^2^ who underwent a robotic-assisted laparoscopic low anterior resection after neoadjuvant chemotherapy for lower rectal cancer. Although temporary diverting ileostomy was initially considered, stoma creation was skipped intraoperatively, considering the complication risk-benefit ratio. Case 2 involved a 42-year-old female patient with a BMI of 64 kg/m^2^ who underwent open partial non-curative colon resection for descending colon cancer complicated by colonic perforation and abscess formation. The patient was considered to be at high risk of stoma-related complications due to high mobility of the subcutaneous fat of abdominal wall; therefore, we decided not to create a stoma preoperatively.

**Discussion:**

Considering the high risk of stoma-associated complications, avoiding stoma creation and implementing preventive measures against potential complications are alternative options for patients with super-morbid obesity.

**Conclusion:**

We present our experience two cases in which stoma creation was avoided for super-morbid obese patients with BMI over 40.

## Highlights

• Stoma creation is performed in patients with a high risk of anastomotic leakage.
• There are not a few risks associated with stoma in super-morbid obese patients.
• We presented two cases of avoidance of stoma creation in super-morbid obese patients.
• Methods other than stoma creation might be considered in super-morbid obese patients.

## 1 Introduction

Stoma creation is performed in patients with a high risk of anastomotic leakage [1]. Previous studies have reported inconsistent findings regarding the incidence of complications associated with stoma creation, which varies from 10 % to 70 % [[2], [3], [4]]. Stoma creation has not just aesthetic drawbacks that would adversely affect the quality of life of patients [5].

An appropriate length of the intestine must be maintained to penetrate the thick subcutaneous fat during stoma creation, particularly in patients with obesity who also have usually; however, due to thick mesenteric fat, lifting out the intestine from the abdominal cavity while maintaining blood flow is technically challenging. Consequently, stoma-related complications are more frequent in such patients, highlighting the need for carefully considering the indications for stoma creation [6,7].

Here, we present two cases in which, despite being indicated, stoma creation was avoided because of the high risk of stoma-related complications due to super-morbid obesity. Our work has been reported in line with the SCARE Guidelines 2020 criteria [8].

## 2 Presentation of case

**Fig. 1:**
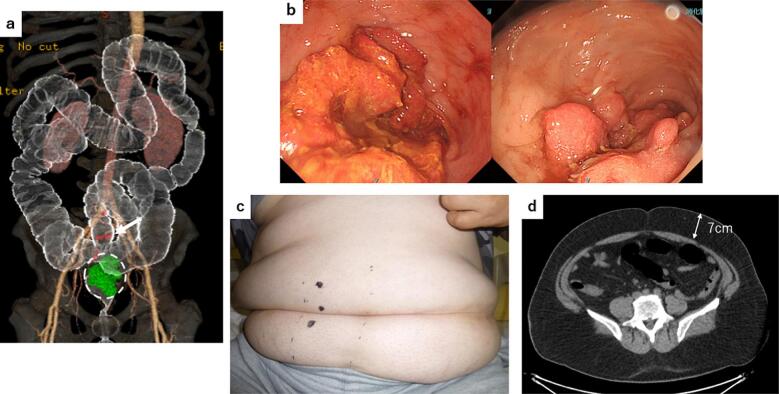
(a) In 3D-CT, the primary site of rectal cancer is indicated by a green tumor (circle), and lymph node metastases are indicated by a red node (arrow). (b) Pre-chemotherapy colonoscopy shows a semi-peripheral type 2 lesion in the rectum (left), and post-chemotherapy colonoscopy shows shrinkage from pre-chemotherapy (right). (c) Preoperative stoma site markings. (d) Subcutaneous fat thickness at the umbilical level is 7 cm. (For interpretation of the references to colour in this figure legend, the reader is referred to the web version of this article.)

Case 1: A 52-year-old male patient (height, 169.5 cm; weight, 120 kg; and body mass index (BMI), 41.8 kg/m^2^) presented with positive fecal occult blood test results. Further examination revealed lower rectal cancer with lymph node metastases, cT3N2aM0, cStage IIIB (Union for International Cancer Control (UICC) 8th edition) (Fig. 1a, b left). Since he had several comorbidities including diabetes and hypertension, and was considered to be a high-risk patient for postoperative complications due to severe obesity, we decided to administer preoperative chemotherapy (capecitabine plus oxaliplatin, four courses) and attempted to reduce his weight during that period; the patient lost 9.3 % of body weight, which was reduced to 112 kg. Colonoscopy performed after chemotherapy revealed shrinkage of the primary tumor, and the endoscopic response was considered to be partial (Fig. 1b right). Robotic-assisted low anterior resection was selected as the surgical method. The tumor was located 6 cm from the anal verge, and temporary diverting ileostomy was initially planned, given the high risk of anastomotic leakage associated with severe obesity. Stoma site marking was performed (Fig. 1c). Intraoperative findings revealed intestinal malrotation and extensive intra-abdominal adhesions with thick mesenteric fat. Additionally, preoperative computed tomography (CT) revealed 7-cm subcutaneous fat at the umbilical level (Fig. 1d). If temporary diverting ileostomy was to be created, it would have been difficult to ensure intestinal blood flow, potentially leading to stoma necrosis and an increased risk of postoperative stoma-related complications. Although the anastomosis site was 3 cm from the anal verge, the air leak test result was negative, and the risk of anastomotic leakage was not substantially high. Therefore, stoma creation was skipped, and a silicone transanal drainage tube was placed. Because of the high risk of anastomotic leakage, the postoperative fasting period was extended by 6 days to determine the absence of leakage. The postoperative course was uneventful except for minor complications such as surgical site infection (SSI), and the patient was discharged on the 27th postoperative day.

**Fig. 2:**
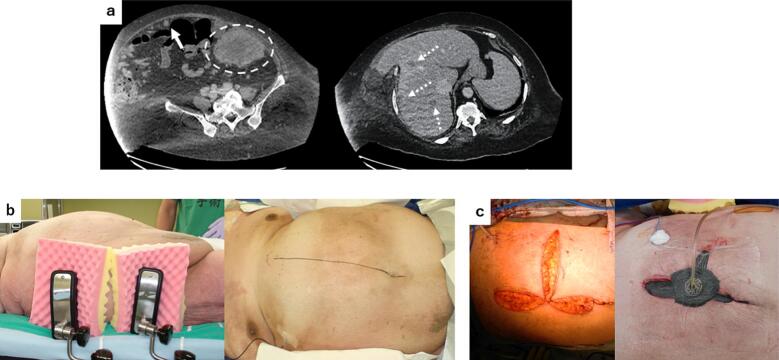
(a) CT image showing the primary descending colon cancer (left circle), a nodule suspected of peritoneal dissemination (left arrow) and liver metastases (right arrow). The thickness of the subcutaneous fat could not be measured because the thickness of the abdominal wall exceeded the CT imaging range. (b) Preoperative simulation photograph. The abdominal wall was fixed with a side plate owing to the large amount of subcutaneous fat that caused it to sag. (c) Epidermal closure was not performed (left) and NPWT was initiated (right).

Case 2: A 44-year-old female patient (height, 155 cm; weight, 153 kg; and BMI, 64 kg/m^2^) presented with fever and poor appetite. Detailed examinations revealed descending colon cancer, cT4aN2aM1c (H2, PUL2, P3), cStage IVC (UICC 8th edition) with colonic penetration and abscess formation (Fig. 2a). In this patient, distant metastases were present, curative resection was not possible, and early initiation of systemic chemotherapy was necessary. Surgery was planned to control the intra-abdominal localized sepsis or infection as it is not just inflammation with abscess present and enable oral intake. Because she had obesity-related cardiopulmonary comorbidities, we decided not to perform intraoperative body repositioning that would alter circulatory dynamics. On the day before the surgery, we performed positional simulation in the operating room and confirmed its safety (Fig. 2b). Various surgical approaches, such as transverse colostomy or Hartmann's procedure, were initially considered. However, substantial mobility of a large amount of subcutaneous fat was observed from the epigastric to the femoral region. In case of stoma creation, compression of the stoma by massive fat would be highly likely, and the risk of stoma-related complications was considered to be very high. Therefore, one-stage colectomy and anastomosis were performed. An open approach was used to minimize the impact on cardiopulmonary function. The peritoneal disseminated nodules firmly infiltrated the area surrounding the umbilicus; therefore, a transverse incision was made to excise them because wound healing failure was inevitable if it was not removed. Anastomosis was performed in non-inflamed and non-edematous areas and silicone transanal drainage tube was placed. SSI was deemed inevitable, skin closure was not performed (Fig. 2c left) for early initiation of negative pressure wound therapy (NPWT) postoperatively (Fig. 2c right). Because of the high risk of anastomotic leakage, the postoperative fasting period was extended by 9 days to determine the absence of leakage. No severe significant complications occurred except for SSI, and the patient was discharged on the 40th postoperative day.

## 3 Discussion

We reported two cases of patients in whom stoma creation was avoided. In Case 1, based on the intraoperative assessment, the patient had thick mesenteric fat, and the risk of anastomotic leakage was not substantially high. However, considering as compared with the potential risk of postoperative stoma-related complications, intraoperative stoma creation was avoided. In Case 2, the patient had thick subcutaneous fat as well as mesenteric fat, with high abdominal wall mobility, suggesting a high risk of stoma-related complications, such as stoma retraction. Therefore, we decided not to create a stoma preoperatively. In patients with obesity, no consensus has been reached on stoma creation. It is also known that obese patients are at higher risk of anastomotic leakage, especially in patients with inflammation such as case 2, which may be accompanied by edematous changes in the intestinal tract, so further caution is needed [9]. Once suture failure occurs, especially in super-morbid obese patients, it is more difficult to access the site of anastomotic leakage than in non-obese patients. In this regard, the risk of anastomotic leakage should be considered as great as possible. Therefore, its indications should be carefully considered and based on preoperative and intraoperative findings, prediction of subsequent risks and benefits.

In general, colorectal surgery in patients with obesity is associated with prolonged operative time, increased blood loss, and a higher incidence of surgical complications such as anastomotic leakage, wound infection, and lower extremity deep vein thrombosis than that in patients without obesity [[10], [11], [12], [13]]. To reduce the risk of complications, countermeasures such as insertion of a transanal drainage tube [14], use of NPWT [15], and antithrombotic therapy are adopted. Considering the availability of time, preoperative weight loss can mitigate the risk of complications. In Case 1, the weight of the patient was reduced by 9.3 % during preoperative chemotherapy, and in Case 2 NPWT were performed, and both Cases, transanal drainage tube was placed as countermeasures against postoperative complications. In addition, it is generally reported that early resumption of oral intake is helpful in avoiding early postoperative complications [16], but in our cases, we extended the fasting period. The reason for this measure was that fasting is a conservative treatment for anastomotic leaks [17], and considering the risk of early anastomotic leakage, we judged that it was safer to perform oral intake after confirming passage of gastrointestinal tract such as exhaust gas. Both patients were discharged without major complications. In both cases, however, the length of hospital stay was longer, at 27 and 40 days. This is due to the insurance system in Japan, which generally requires longer hospital stays than in Western countries [18]. This was not only due to problems with the insurance system, but also due to the lack of financial and social support, and the patients was discharged after their condition had fully recovered. In cases of severe obesity, preoperative simulation is important to ensure safe patient positioning during surgery. In both the cases in our study, careful preoperative simulation was performed in the operating room on the day before surgery, and no intraoperative complications in patient positioning and fixation occurred.

In patients with obesity, careful consideration is necessary when determining the indications for a stoma due to the incidence of stoma-related complications reported as 66 %, significantly higher than in non-obese patients [19], so careful consideration should be given when deciding on the indication for a stoma in obese patients. One reason for this is the thickness of subcutaneous fat. Generally, the thicker the subcutaneous fat, the longer the intestinal length required to lift it out of the body. Therefore, patients with obesity require a longer intestinal tract than patients with standard body weight, and stoma creation often requires longer intestinal tracts than anastomosis, indicating the need for a wider range of mobilization [6]. Intestinal lifting is typically achieved by passing the intestine through the rectus abdominis muscle to prevent peristomal hernias or stoma prolapse [20]. However, in patients with obesity, visualization of the rectus abdominis muscle is difficult because of the thick abdominal wall. Thus, we must consider that patients with obesity have a higher risk of postoperative complications, including stoma-related complications, even if their bowel length is adequate. Furthermore, a large amount of subcutaneous fat is heavy, causing the abdominal wall to droop with gravity, and has very high mobility [6]. The excessive tension in the lifted intestine may lead to stoma retraction. In addition, a large abdominal wall may render stoma visualization difficult, leading to challenging stoma management and an increased risk of management-related complications [6,21]. Another reason is the thick mesenteric fat. In patients with a standard body weight, visualizing blood vessels in the mesenteric fat and performing mesenteric procedures while ensuring blood flow to the intestine is not difficult. However, patients with obesity have massive mesenteric fat, and visualization of the vessels within the mesentery is often difficult, making the surgical procedure considerably more challenging. Mishandling of the blood vessels in the mesentery can lead to ischemic intestinal necrosis, requiring additional surgery. Therefore, great care should be taken when handling the mesentery in patients with obesity. Even if sufficient intestinal length and blood flow are ensured during mesenteric preparation, lifting the intestine out of the body can be difficult due to the large amount of mesenteric fat. Even if it can be lifted, if the diameter of the intestine is too large for an incision in the abdominal wall, blood flow will be obstructed, leading to stomal necrosis. Previous reports showed that the typical situation in which a colostomy becomes difficult occurs during emergency surgery in obese men with a short, thickened mesentery and a very thick abdominal wall, in which a primary anastomosis may be safer [22,23]. Moreover, careful handling should be performed when enlarging the laparotomy to lift the bowel, as this increases the risk of parastomal hernia or stoma prolapse [21,24,25]. In the two cases reported in our study, we considered that stoma-related complications were inevitable because of the thick subcutaneous fat with high mobility and the thick mesenteric fat, leading to the decision to avoid stoma creation.

In conclusion, avoiding stoma and implementing countermeasures to prevent potential complications could be an alternative option in cases where stoma creation has a high risk.

## Sources of funding

No funding or grant support.

## Ethical approval

Ethical approval is not applicable. The case report is not containing any personal information.

## Registration of research studies

Not applicable.

## Consent

Written informed consent was obtained from the patient for publication and any accompanying images. A copy of the written consent is available for review by the Editor-in-Chief of this journal on request.

## Author contribution

Naoto Fujimoto drafted the manuscript. Takayuki Ogino helped with finalizing the manuscript, and Hidetoshi Eguchi gave the final approval of the article. All authors have read and approved the final manuscript.

## Guarantor

Takayuki Ogino.

## Declaration of competing interest

There are no conflicts of interest to declare by all the authors.
